# A rapid stability-indicating, fused-core HPLC method for simultaneous determination of β-artemether and lumefantrine in anti-malarial fixed dose combination products

**DOI:** 10.1186/1475-2875-12-145

**Published:** 2013-04-30

**Authors:** Sultan Suleman, Kirsten Vandercruyssen, Evelien Wynendaele, Matthias D’Hondt, Nathalie Bracke, Luc Duchateau, Christian Burvenich, Kathelijne Peremans, Bart De Spiegeleer

**Affiliations:** 1 Drug Quality and Registration (DruQuaR) group, Faculty of Pharmaceutical Sciences, Ghent University, Harelbekestraat 72, Ghent, B-9000, Belgium; 2 School of Pharmacy, Jimma University, P.O. Box 378, Jimma, Ethiopia; 3 Department of Medical Imaging, Physiology and Biometrics, Faculty of Veterinary Medicine, Ghent University, Salisburylaan 133, Merelbeke, B-9820, Belgium

**Keywords:** Anti-malaria, β-artemether, Lumefantrine, Stability-indicating assay, HPLC-UV, Fused-core, Finished pharmaceutical product, Quality-by-design (QbD)

## Abstract

**Background:**

Artemisinin-based fixed dose combination (FDC) products are recommended by World Health Organization (WHO) as a first-line treatment. However, the current artemisinin FDC products, such as β-artemether and lumefantrine, are inherently unstable and require controlled distribution and storage conditions, which are not always available in resource-limited settings. Moreover, quality control is hampered by lack of suitable analytical methods. Thus, there is a need for a rapid and simple, but stability-indicating method for the simultaneous assay of β-artemether and lumefantrine FDC products.

**Methods:**

Three reversed-phase fused-core HPLC columns (Halo RP-Amide, Halo C18 and Halo Phenyl-hexyl), all thermostated at 30°C, were evaluated. β-artemether and lumefantrine (unstressed and stressed), and reference-related impurities were injected and chromatographic parameters were assessed. Optimal chromatographic parameters were obtained using Halo RP-Amide column and an isocratic mobile phase composed of acetonitrile and 1mM phosphate buffer pH 3.0 (52:48; V/V) at a flow of 1.0 ml/min and 3 μl injection volume. Quantification was performed at 210 nm and 335 nm for β-artemether and for lumefantrine, respectively. *In-silico* toxicological evaluation of the related impurities was made using Derek Nexus v2.0®.

**Results:**

Both β-artemether and lumefantrine were separated from each other as well as from the specified and unspecified related impurities including degradants. A complete chromatographic run only took four minutes. Evaluation of the method, including a Plackett-Burman robustness verification within analytical QbD-principles, and real-life samples showed the method is suitable for quantitative assay purposes of both active pharmaceutical ingredients, with a mean recovery relative standard deviation (± RSD) of 99.7 % (± 0.7%) for β-artemether and 99.7 % (± 0.6%) for lumefantrine. All identified β-artemether-related impurities were predicted in Derek Nexus v2.0® to have toxicity risks similar to β-artemether active pharmaceutical ingredient (API) itself.

**Conclusions:**

A rapid, robust, precise and accurate stability-indicating, quantitative fused-core isocratic HPLC method was developed for simultaneous assay of β-artemether and lumefantrine. This method can be applied in the routine regulatory quality control of FDC products. The *in-silico* toxicological investigation using Derek Nexus® indicated that the overall toxicity risk for β-artemether-related impurities is comparable to that of β-artemether API.

## Background

Malaria is endemic throughout most of the tropics where approximately three billion people, living in 108 countries, are exposed. Approximately 243 million people annually develop symptomatic malaria [1]. Most of these can be attributed to *Plasmodium falciparum*, but *Plasmodium vivax* and *Plasmodium knowlesi* can also cause severe diseases. An estimated 3.3 billion people were at risk of malaria in 2010 with populations living in sub-Saharan Africa having the highest risk of acquiring malaria, and children under five years of age and pregnant women being most severely affected [2,3]. Malaria case management remains a vital component of malaria control strategies. This entails early diagnosis and prompt treatment with effective anti-malarial medicines [4]. The World Health Organization (WHO) has recommended that all anti-malarials should consist of a combination of an artemisinin derivative with a co-drug such as lumefantrine, amodiaquine or mefloquine; most malaria endemic countries have now adopted artemisinin-based anti-malarial combination therapy (ACT) as first-line treatment of *P. falciparum* malaria in place of chloroquine, quinine and sulphadoxine-pyrimethamine fixed dose combinations [5]. However, the emergence of resistance is of great concern [6-8], and this problem is fuelled by poor quality anti-malarial drugs.

Poor quality anti-malarials are a severe under-recognized public health problem, reducing the effectiveness of these drugs and threatening current treatment policies [9]. There are three main types of poor quality medicines: substandard, degraded and counterfeit. Substandard drugs are produced with inadequate attention to good manufacturing practices and may have content outside accepted limits. Degraded formulations may result from (unwanted) exposure of initially well produced, good quality medicines to light, heat and humidity [10,11]. Therefore, the ultimate purpose of stability testing is to provide evidence on how the quality of a drug varies with time under the influence of a variety of environmental factors such as temperature, humidity and light and enables recommendations of storage conditions, retest periods and shelf life to be established. The two main chemical aspects of the drug product that play an important role in shelf-life determinations are the assay of active drug (efficacy) and degradants generated during the stability study (safety). The assay of drug product in stability test samples obviously needs to be determined using a stability-indicating method, as recommended by the International Conference on Harmonization (ICH) guidelines [12,13]. Moreover, the intrinsic stability of a finished drug product should also be considered as a possible quality attribute when evaluating and comparing different drug products with the same active pharmaceutical ingredient (API). For example, it has been demonstrated that the half-life of β-artemether-containing products at 50°C can range between 0.70 and 9.52 months [14].

**Figure 1:**
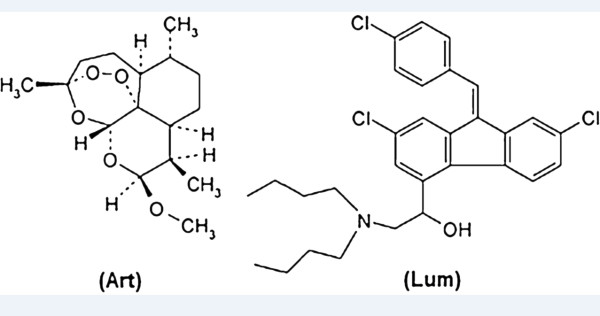
Structure of β-artemether (Art) and lumefantrine (Lum).

β-artemether is a methyl ether derivative of artemisinin, which is a peroxide lactone isolated from the Chinese anti-malarial plant *Artemisia annua* (Figure 1). Chemically, it is (+)-(3-alpha,5a-beta,6-beta,8a-beta,9-alpha,12-beta,12aR)-decahydro-10-methoxy-3,6,9-trimethyl-3,12-epoxy-12H-pyrano(4,3-j)-1,2-benzodioxepin [15]. Lumefantrine (benflumetol) is a 2,4,7,9-substituted fluorene (2,3-benzindene) derivative (Figure 1). Chemically, it is (9Z)-2,7-dichloro-9-[(4-chlorophenyl)methylene]-a-[dibutylamino) methyl]-9*H*-fluorene-4-methanol [16]. Both compounds are now commercially available in fixed combination products (ACT), which are proven to be highly efficacious for treatment of uncomplicated *P. falciparum* malaria. The increasing use of these β-artemether-lumefantrine combination anti-malarial products and the intrinsic stability of these products requires controlled storage conditions. However, in resource-limited settings, stability of these products is not guaranteed since the supply chains do not have consistently appropriate temperature and humidity quality assurance systems [17]. Therefore, it is important to have a rapid, but robust and stability-indicating quantitative method for the simultaneous assay of β-artemether and lumefantrine in fixed dose combination (FDC) products.

Currently, there are HPLC methods for the assay of β-artemether in finished pharmaceutical products (FPP) [18-20], as well as for lumefantrine analysis [21-23]. Only a few HPLC methods were reported for the quantitative determination of β-artemether and lumefantrine in fixed combination anti-malarial products [24-26]. However, no simple, rapid and proven stability-indicating method has been reported for the simultaneous analysis of both active pharmaceutical drug products. Hence, the present study reports a rapid, economical, precise and accurate method for the assay of β-artemether and lumefantrine in the presence of their degradants.

## Methods

### Samples and chemicals

β-artemether and lumefantrine APIs, Co-Artesiane® FPP powder for oral suspension, dihydroartemisinin (DHA), artemisinin, 9,10-anhydroartemisinin (AHA; late eluting impurity (LEI)) and α-artemether standards were supplied by Dafra Pharma International (Belgium). Coartem® and Artemine® samples were collected from different markets in Ethiopia. Analytical solutions were prepared using HPLC grade unstabilized tetrahydrofuran (THF) (Fisher Scientific, Leicestershire, UK) obtaining a concentration of 0.2 mg/ml β-artemether and 1.2 mg/ml lumefantrine corresponding to 100% label claim (lc). Hydrogen peroxide (H_2_O_2_) and sodium hydroxide (NaOH) were purchased from Merck (Darmstadt, Germany), hydrochloric acid (HCl) and o-phosphoric acid from Sigma-Aldrich (St Louis, USA). Sartorius (Göttingen, Germany) ultrapure 18.2 mΩ.cm quality water and HPLC grade acetonitrile (Fisher Scientific, UK) were used for HPLC-UV analysis.

### Liquid chromatography and analytical conditions

The HPLC analyses were carried out using HPLC-PDA apparatus consisting of a Waters Alliance 2695 separation module and a Waters 2998 photodiode array detector with Empower 2 software for data acquisition (all Waters, Milford, MA, USA). The following fused-core stationary-phase chemistries were evaluated: (i) Halo RP-Amide (50×4.6 mm id; 2.7 μm particle size); (ii) Halo C18 (50×4.6 mm id; 2.7 μm particle size); and, (iii) Halo Phenyl-hexyl (50×4.6 mm id; 2.7 μm particle size), all Achrom (Machelen, Belgium) and all thermostated at 30°C. Detection was performed from 190–400 nm. Lumefantrine quantification was done at 335 nm, a wavelength at which β-artemether and its related impurities are not absorbing. For β-artemether, quantification was performed at 210 nm. As N-oxide-lumefantrine might interfere if present, back-calculating the peak area of N-oxide-lumefantrine using peak area conversion factor from 335 nm to 210 nm (1.64) was performed and the obtained value was subtracted from the peak area at 210 nm. The injection volume was 3 μl. Isocratic mobile phases containing acetonitrile and 1 mM phosphate buffer pH 3.0 were used at a flow of 1.0 ml/min. The separation of β-artemether and lumefantrine was evaluated using different proportions of these mobile phase solvents and, for each condition, the retention factor (*k’*) and asymmetry factor (A_s_) were calculated based on the method described in European Pharmacopoeia (Ph. Eur.) 2.2.46 [27] (see Additional file 1). The optimal condition was achieved using the Halo RP-Amide column and a mobile phase composed of acetonitrile and 1 mM phosphate buffer pH 3.0 (52:48 V/V).

### Preparation of solutions

#### *Preparation of β-artemether and lumefantrine standard solution*

Approximately 20.0 mg β-artemether and 120.0 mg lumefantrine reference standards were accurately weighed and transferred to a 100.0 ml volumetric flask. Eighty ml tetrahydrofuran was added to dissolve both compounds and the solution was diluted to volume using mobile phase.

#### *Preparation of test sample solutions*

Four samples of fixed dose combination tablets (Coartem® and Artemine®) containing 20 mg β-artemether and 120 mg lumefantrine and three powders for oral suspension stability samples (Co-artesiane®) containing 180 mg β-artemether and 1080 mg lumefantrine were analysed using the validated fused-core HPLC method. For this, a homogenous FPP powder amount equivalent to 20.0 mg β-artemether and 120.0 mg lumefantrine was accurately weighed and transferred to a 100.0 ml volumetric flask. Eighty ml tetrahydrofuran was added, shaken for 5 min and diluted to volume using mobile phase. The mixture was filtered through 0.45 μm HPLC syringe filters and analysed using HPLC.

### Preparation of stress solutions

#### *Preparation of oxidative degradation of lumefantrine API solution*

Approximately 120.0 mg lumefantrine API was accurately weighed and transferred into 100 ml Erlenmeyer flask; 45.0 ml tetrahydrofuran was added to ensure complete dissolution of lumefantrine and then 5.0 ml 30% hydrogen peroxide was added. The solution was boiled for 120 min under constant reflux and analysed using HPLC.

#### *Preparation of acidic degradation of lumefantrine solution*

Approximately 120.0 mg of lumefantrine API was accurately weighed into 100 ml Erlenmeyer flask; 10.0 ml of 1 M hydrochloric acid solution was added and incubated at 70°C for 30 hours. Subsequently, the solution was neutralized by addition of 2.0 ml of 5 M sodium hydroxide solution and then 38.0 ml of THF was added. The mixture was sonicated for 5 min, filtered and analysed.

#### *Preparation of heat stressed β-artemether API solution*

Preparation of heat stressed β-artemether was performed as described by De Spiegeleer *et al*. [20]. Briefly, approximately 20.0 mg of β-artemether API was accurately weighed and transferred into a glass HPLC vial. The vial was put in a heating block at 145°C for 30 min, resulting in approximately 70% conversion of β-artemether to related degradation products. Then 1.0 ml tetrahydrofuran was added and the solution was quantitatively transferred to a 50.0 ml volumetric flask by addition of 40 ml tetrahydrofuran. The solution was then diluted to volume using mobile phase.

### Validation

#### *Linearity*

A stock solution containing 250 μg/ml β-artemether and 1,500 μg/ml lumefantrine in THF was prepared in triplicate. Different aliquots of these solutions were diluted in a dilution solvent consisting of THF/mobile phase (80:20 V/V) to five different concentrations, corresponding to 160, 180, 200, 220 and 240 μg/ml of β-artemether, and 960, 1,080, 1,200, 1,320 and 1,440 μg/ml of lumefantrine. Calibration curves for concentration *versus* peak area were plotted for each compound and the obtained data were subjected to linear regression analysis.

#### *Precision*

For intra-day precision, six sample solutions (n=6) were prepared at 0.2 mg/ml β-artemether and 1.2 mg/ml lumefantrine concentrations and analysed using HPLC. Similarly, the inter-day precision was evaluated in three consecutive days (n=3×6). β-artemether and lumefantrine concentrations were determined and relative standard deviations (RSD) were calculated.

#### *Accuracy (recovery test)*

Accuracy was tested by recovery experiments where β-artemether and lumefantrine reference solutions were added to a placebo sample at three levels: 75%, 100% and 125% of the label claim. At each level, samples were prepared in duplicate and recovery percentage was calculated.

#### *Selectivity*

Selectivity of the method was evaluated by injecting the stressed β-artemether and lumefantrine solutions as well as reference standard solutions of α-artemether, artemisinin, DHA and AHA. Moreover, UV-spectral purities of β-artemether and lumefantrine chromatographic peaks were evaluated using Waters’ peak purity PDA evaluation.

#### *Robustness*

A Plackett-Burman experimental design consisting of 12 experiments with two replicates in block was used for the robustness testing (Modde version 8, Umetrics Inc, USA). Three sample solutions (stressed, test sample and reference solutions) were prepared at 100% lc and analysed using different experimental conditions by varying different analytical parameters: flow (0.8, 1.0, and 1.2 ml/min), acetonitrile proportion (50%, 52% and 54%), mobile phase pH (2.8, 3.0 and 3.2), and column temperature (25°C, 30°C and 35°C). β-artemether and lumefantrine contents and different chromatographic characteristics were determined under each condition.

#### *Limit of detection (LoD) and limit of quantitation (LoQ)*

Combined standard solutions of β-artemether and lumefantrine were prepared by serial dilutions, ranging from 0.4 to 25 μg/ml for β-artemether and 0.2 to 11.5 μg/ml for lumefantrine, and injected onto the chromatographic system. The LoD was defined as the concentration for which a signal-to-noise ratio (S/N) of three was obtained and LoQ was considered to be the concentration at which S/N was 10.

#### *In-silico toxicological predictions*

*In-silico* toxicological study for lumefantrine and its related impurities was reported in previous publication [23]. Exhaustive impurity profiling of β-artemether (including its possible degradants) was also reported [20,28]. Therefore, to make *in-silico* toxicological comparative predictions for β-artemether and its identified related impurities, Derek Nexus v2.0 for Windows developed by Lhasa Ltd (Leeds, UK) was used. Derek Nexus® is an expert knowledge-based system, containing descriptions of molecular substructures which have been associated with toxic endpoints (structural alerts), that predicts a probability whether a chemical is toxic in humans, other mammals and bacteria. The program applies structure-activity relationships [(Q)SARs] and expert knowledge rules to derive a reasoned conclusion about the potential toxicity of the query chemical [29-31].

## Results

### Development

**Figure 2:**
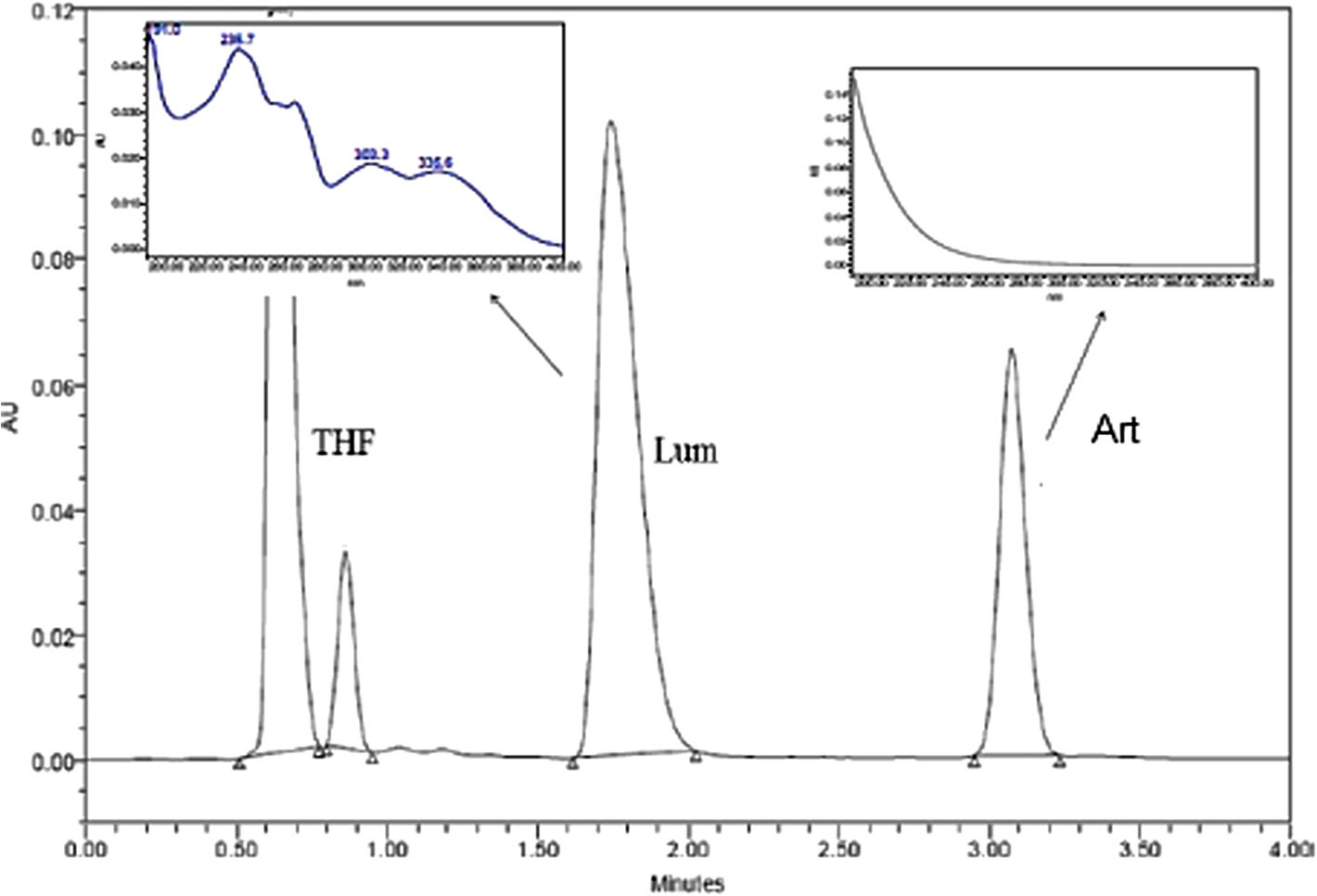
Typical chromatogram obtained on solution of β-artemether (Art) (RT: 3.07min) and lumefantrine (Lum) (RT: 1.70 min) in tetrahydrofuran (THF) with their UV spectrum in the infronts.

To develop a rapid, simple and stability-indicating isocratic HPLC method, three different fused-core stationary phases (Halo phenyl-hexyl, Halo C18 and Halo RP-Amide) and a mobile phase with different compositions of acetonitrile and 1 mM phosphate buffer with varying pH (3.0, 5.0 and 7.0) were used. Relatively longer run time was obtained with 1 mM phosphate buffer pH 7.0 while pH 5.0 resulted in poor peak shape for lumefantrine. At all conditions, there was no separation between β-artemether and lumefantrine using Halo phenyl-hexyl stationary phase. Using Halo C_18_ stationary phase column and a mobile phase composed of acetonitrile and 1 mM phosphate buffer pH 3.0, the retention factors obtained for β-artemether and lumefantrine were 11.8 and 3.0, respectively. Under these conditions, in spite of achieving good separation between β-artemether and lumefantrine, the peak shape of lumefantrine was found to be out of pharmacopoeial specifications (Ph Eur. specification A_s_ ≤ 1.5) [27] and the total run time was relatively long, i e, 6 min. Substituting the Halo C_18_ with a Halo RP-Amide stationary phase, different proportions of mobile phase solvents were evaluated (Table 1). The optimal mobile phase, composed of acetonitrile and 1 mM phosphate buffer pH 3.0 (52:48, V/V), gave an adequate retention factor *k*’ and lumefantrine peak shape (A_s_ 1.3) that complies with pharmacopoeial specifications within a short period of total run time of 4 min (Figure 2).

**Table 1:** Chromatographic parameters for β-artemether and lumefantrine at different mobile phase compositions using Halo RP-Amide stationary phase column

**Mobile phase composition; Acetonitrile: 1mM phosphate buffer pH 3.0**	**β-artemether retention factor (*k’*)**	**β-artemether peak symmetry factor (A_s_)**	**Lumefantrine retention factor (*k’*)**	**Lumefantrine peak symmetry factor (A_s_)**
54:46	5.5	1.0	1.5	1.4
52:48	6.8	1.0	2.0	1.3
50:50	7.5	1.0	2.8	1.8

*t*_*o*_ = 0.4 min.

As indicated in the UV-spectra of Figure 2, β-artemether only shows reasonable UV-absorption at the lower wavelengths of the spectrum (200-230 nm), due to the absence of UV-chromophores in its structure. Thus, its quantification was performed at 210 nm. For lumefantrine with a RT of 1.70 min, quantification was performed at 335 nm, the wavelength at which no UV-absorption interference from β-artemether was observed.

It was reported that the desbenzyl keto derivative (DBK) is the major degradation product after acidic degradation of lumefantrine, while the oxidative degradation of lumefantrine was reported to yield N-oxide-lumefantrine [23]. Under the chromatographic conditions, DBK eluted before lumefantrine at retention time (RT) of 1.26 min, while N-oxide-lumefantrine was eluting after lumefantrine at RT of 3.17 min (see Additional file 1).

As indicated in Table 2, β-artemether and all its identified related impurities (α-artemether, DHA, artemisinin and AHA) were eluting at different RT without any interference with the main peak. Previous studies have indicated that AHA-β-artemether is the most critical pair to be separated [28], while other impurities like DKA or furano acetate are sufficiently well separated from β-artemether. Moreover, lumefantrine and its related degradation impurities (DBK and the N-oxide of lumefantrine) were also eluting at different RT. However, while some of β-artemether impurities (DHA, artemisinin and other degradation products from dry heat stress β-artemether) co-elute with lumefantrine, which however does not interfere with its assay due to negligible UV-absorption of these β-artemether impurities at 335 nm, the N-oxide-lumefantrine problematically co-elutes with β-artemether peak, making selective quantification of β-artemether at 210 nm difficult. Therefore, the method quantifies lumefantrine separated from its related impurities at 335 nm (where β-artemether and its related impurities are not absorbing). Since lumefantrine and its related impurities have strong UV absorption at 210 nm and N-oxide-lumefantrine is co-eluting with β-artemether, it is possible to selectively obtain the UV_210 nm_ peak area of β-artemether alone by back-calculating the peak area of N-oxide-lumefantrine using the peak area conversion factor from 335 nm to 210 nm (*i.e*, 1.64) and subtracting the value from the co-eluting peak area at 210 nm.

**Table 2:** Retention time (RT) for β-artemether and lumefantrine and their related impurities

**#**	**β-artemether**	**DHA**	**Artemisinin**	**α-artemether**	**AHA**	**Lumefantrine**	**DBK**	**N-oxide of lumefantrine**
RT (min)	3.07	1.62	1.68	2.14	2.71	1.70	1.26	3.17

Therefore, compared to the method described in Ph. Int. [26], which uses conventional HPLC, the developed fused-core method uses simple sample extraction technique and is isocratic, rapid, less costly and stability-indicating.

### Validation

#### *Linearity*

Almost all the variation in peak area was explained by the linear concentration (0.9997 for β-artemether and 0.9997 for lumefantrine), indicating the linearity of the method in the assayed range (80 to 120% label claim). The regression analysis data are presented in Table 3.

**Table 3:** Calibration curve for β-artemether and lumefantrine

**Regression parameters**	**β-artemether**	**Lumefantrine**
Regression coefficient, R^2^	0.9997	0.9997
Slope ± standard error	103.1±1.1	4880.8±51.8
Intercept ± standard error	1708.1±235.7	224820.8±62658.3
Relative standard error (%)	1.1	1.1
Concentration range (μg/ml)	160-240	960-1440
F-value	8060.4	8876.5
Number of points	5	5

#### *Precision*

In the prepared solutions for analysis, 100% label claim (lc) represents 0.2 mg/ml β-artemether and 1.2 mg/ml lumefantrine solution.

#### *Intra-day precision*

Mean contents and RSD of β-artemether and lumefantrine in the intra-day precision analysis (n=6) were 99.6% lc with RSD = 1.2% and 99.2% lc with RSD = 0.5%, respectively.

#### Inter-day precision

Mean contents and RSD values of β-artemether and lumefantrine in the inter-day precision analysis (n=3×6) were 99.6% lc with RSD = 1.1% and 99.4% lc with RSD = 0.6%), respectively.

For both compounds, the intra-day and inter-day precision % RSD values were lower than 2.0%, revealing precision of the method [32].

#### *Accuracy (recovery test)*

The recovery test was performed by analysing a spiked placebo. β-artemether mean recovery (n=6) was 99.7% (RSD = 0.7%) and lumefantrine mean recovery was 99.7% (RSD = 0.6%), indicating the accuracy of the method.

#### *Selectivity*

The chromatograms obtained with the stressed lumefantrine API solutions showed degradation impurity peaks separated from the main API peak, and similar findings were observed for the stressed β-artemether solutions. Acid-stressed lumefantrine resulted in DBK eluting before lumefantrine at RT of 1.26 min, while oxidative stress resulted in N-oxide-lumefantrine eluting after lumefantrine at RT of 3.17 min. β-artemether and all its identified related impurities (α-artemether, DHA, artemisinin and AHA) were eluting at different RT without any interference with the β-artemether peak. However, at 210 nm, N-oxide-lumefantrine was co-eluting with β-artemether and some degradants of β-artemether were co-eluting with lumefantrine. There is no interference from β-artemether and its impurities for the estimation of lumefantrine and its related impurities at 335 nm. Therefore, for the quantification of the two APIs, the method uses two wavelengths, 210 nm for β-artemether and 335 nm for lumefantrine.

The peak purity indices for both β-artemether and lumefantrine in different marketed FDC anti-malarial drug sample solutions determined with PDA detector under optimized chromatographic conditions indicated that the purity angle for both APIs was less than the purity threshold, revealing no significant excipient interference.

#### *Robustness*

A Plackett-Burman design was used to test the robustness of the method. Plackett-Burman design is a two level fractional factorial design where main effects are heavily confounded with two factor interactions. It is selected for robustness evaluation since it combines less experimentation with maximal information acquisition in the most efficient way.

Four factors, with deliberate small deviations from the method settings, were considered: percentage V/V of acetonitrile in mobile phase (from 50 to 54%), flow (from 0.8 to 1.2 ml/min), pH (from 2.8 to 3.2) and column temperature (from 25 to 35°C) (see Additional file 1).

Mobile phase pH significantly affects the peak shape of lumefantrine while it did not reveal prominent influence on that of β-artemether. Thus, lumefantrine peak symmetry was selected as a critical quality parameter for the robustness test. The final method provided lumefantrine peak shape (A_s_ 1.3) that complies with pharmacopoeial specifications. Moreover, even the deliberate method variations provided better lumefantrine peak shapes (A_s_ 1.4 and 1.8) (Table 1) than the very tailed lumefantrine peak shape (A_s_ 2.1) reported in the literature [23]. In the stressed sample solutions, there was no difference in selectivity between the results of the method setting and the deliberate variations of both β-artemether and lumefantrine APIs and their respective degradation products.

**Figure 3:**
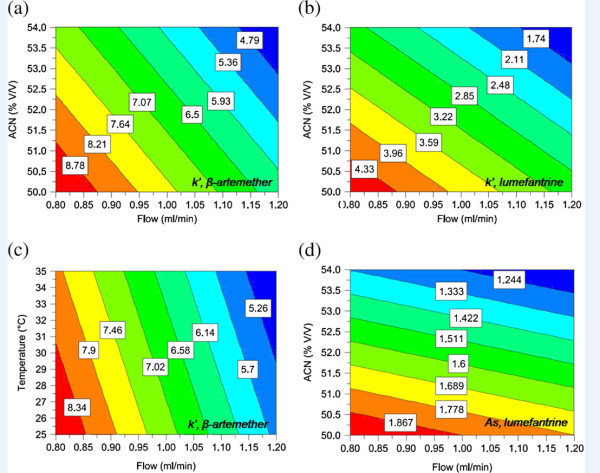
**Contour plots: (a) Acetonitrile (ACN) (% V/V) *vs *flow (ml/min) for retention factor (*k’*) of β-artemether; (b) ACN (% V/V) *vs *flow (ml/min) for *k’ *of lumefantrine; (c) temperature (˚C) *vs *flow (ml/min) for *k’ of *β-artemether; (d) ACN (% V/V) *vs *flow (ml/min) for A_s_of lumefantrine.** For (**a**), (**b**) and (**d**) mobile phase: pH 3, column temperature: 30°C and for (**c**) % ACN: 52, mobile phase: pH 3.

**Figure 4:**
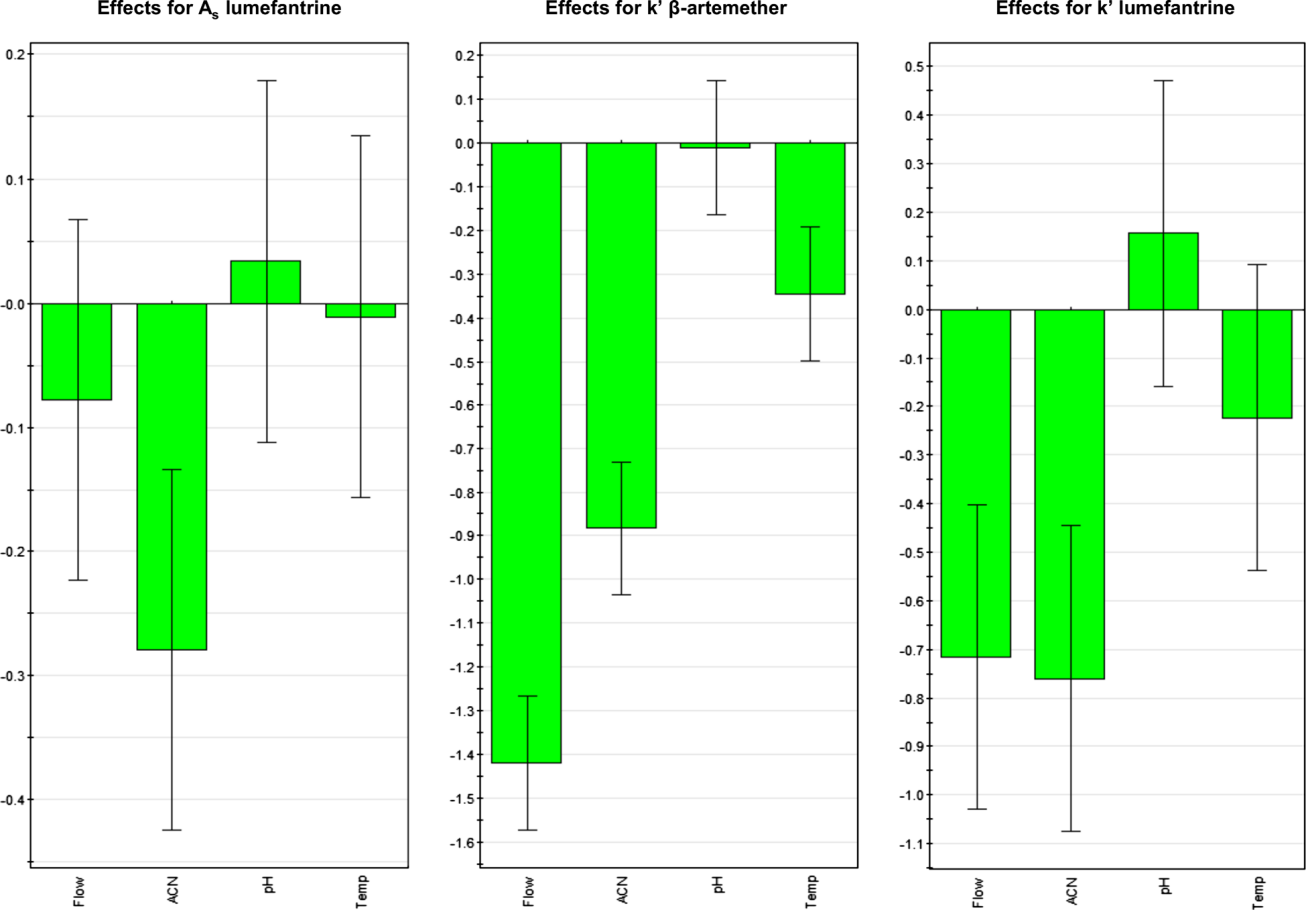
**Observed effects for peak symmetry (A_s_) of lumefantrine and retention factor (*k’*) for β-artemether and lumefantrine in the experimental design space.**

Typical contour plots for different chromatographic parameters as a function of operational variables levels is presented in Figure 3. Figure 3 (a, b and c) is the visual representation of sensitivity, i.e. how quantitatively acetonitrile proportion (%ACN), flow rate and temperature influence the retention factor (*k’*). Moreover, it is revealed in the Figure 3 that small deviations from the method setting introduced in the four parameters do not affect A_s_ and *k’*-specifications set in Ph. Eur. [27]. The observed effects for peak symmetry (A_s_) of lumefantrine and *k’* for β-artemether and lumefantrine are presented in Figure 4. Flow rate and %ACN have more pronounced effect on *k’* of both compounds while lumefantrine peak shape was more affected by %ACN.

The mean content of β-artemether and lumefantrine was found to be 100.9% lc ± 1.0 (RSD 1.0%) and 99.7% lc ± 0.4 (RSD 0.4%), respectively (see Additional file 1). Therefore, the deliberate changes from the method settings in chromatographic conditions (% V/V of acetonitrile in mobile phase (from 50 to 54%), flow (from 0.8 to 1.2 ml/min), pH (from 2.8 to 3.2) and column temperature (from 25 to 35°C)) have little impact on the assay of β-artemether and lumefantrine containing anti-malarial FDC products indicating the robustness of the method.

#### Limit of detection (LoD) and limit of quantitation (LoQ)

According to the determined signal-to-noise ratio, the LoD and LoQ for β-artemether were calculated to be 3.4 μg/ml and 10.0 μg/ml, respectively. For lumefantrine, LoD was 0.1 μg/ml and its LoQ was 0.4 μg/ml. As the purpose of this developed method is to quantitatively determine both β-artemether and lumefantrine simultaneously in FDC anti-malarial products where the compounds exist in the mass ratio β-artemether: lumefantrine of 1:6, the LoD and LoQ values obtained for β-artemether should be considered as the overall detection and quantification limits, while for lumefantrine, the risk of overloading the HPLC system is to be considered. Both opposing aspects are solved with the proposed method.

#### Analysis of marketed FDC products

The results of real sample analysis are presented in Table 4. All the analysed batches presented β-artemether and lumefantrine contents complying with the 95-105% lc specifications. The β-artemether content in the tablet samples varied from 98.2% to 103.2% while lumefantrine content varied from 97.9% to 101.5%. In Co-Artesiane powder for oral suspension FDC product, β-artemether content was in the range of 99.7% to 101.1% while that of lumefantrine was ranging from 100.8% to 102.0%.

**Table 4:** Contents of β-artemether and lumefantrine in fixed dose combination (FDC) products (n=6)

**FDC samples**	**Batch/Lot No.**	**Content (%) ± S.D.**	
		**β-artemether**	**Lumefantrine**
Artemine® tablets	A	103.2 ± 1.5	101.5 ± 0.9
	B	102.2 ± 1.7	101.3 ± 0.5
Coartem® tablets	A	98.2 ± 0.9	97.92 ± 0.7
	B	99.2 ± 1.5	98.8 ± 0.7
Co-Artesiane powder for oral suspension	A	100.9 ± 1.9	101.4 ± 0.9
	B	99.7 ± 1.6	102.0 ± 0.8
	C	101.1 ± 1.3	100.8 ± 0.8

S.D. = standard deviation.

#### *In-silico toxicological predictions of β-artemether and its related impurities*

**Figure 5:**
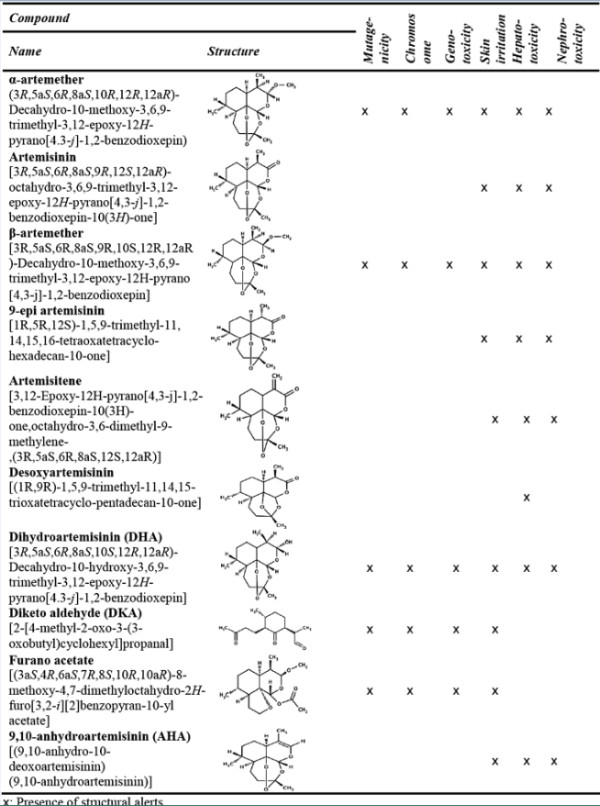
***In-silico *toxicity profile of β-artemether and its impurities.**

*In-silico* toxicity profile of lumefantrine and its related impurities was reported in previous publication [23]. In this study, mutagenicity, chromosome abrasion, genotoxicity, skin irritation, hepatotoxicity and nephrotoxicity endpoints for β-artemether, as well as for its related observed and already described impurities, have been investigated using Derek Nexus® and the result is presented in Figure 5. The toxicity profile of β-artemether and all its identified related degradants and synthetic impurities is defined by several general toxicity alerts. DHA, α-artemether and β-artemether were found to have toxicity endpoints for mutagenicity, chromosomal abrasion, genotoxicity, skin irritation, hepatogenicity and nephrotoxicity. β-artemether and all its identified related impurities, except desoxyartemisinin which has structural alert for hepatotoxicity, have substructures for skin irritation. Derek Nexus® did not trigger mutagenicity, chromosomal abrasion and genotoxicity for artemisinin, 9-epi artemisinin, artemisitene, desoxyartemisinin and AHA.

## Discussion

The development of rapid, economical and reliable stability-indicating methods is essential to assure the quality of anti-malarial drugs in general, and the β-artemether-lumefantrine FDC anti-malarial products in particular. The use of poor quality drugs might contribute to the development of resistance in endemic areas due to the exposition to subtherapeutic doses [19]. The quality control of the anti-malarial pharmaceutical preparations currently on the market might help to assure the efficacy of the treatment and avoid resistance to these anti-malarial drugs.

A simple, rapid and economical stability-indicating, fused-core HPLC method has been developed and validated for the routine quality control tests of β-artemether and lumefantrine in FDC anti-malarial products. The method uses a simple sample preparation procedure (extraction of the APIs with tetrahydrofuran) and is rapid with a total run time of only four minutes. The results of stress testing show that the developed assay method is selective and stability-indicating as it is capable of separating both β-artemether and lumefantrine from their respective degradation products. Moreover, the developed method was successfully applied to quantitatively analyse β-artemether and lumefantrine in marketed FDC anti-malarial drugs.

Safety of a drug product is dependent not only on the toxicological properties of the active drug substance, but also on the toxicological properties of its impurities including the possible degradants. Impurity profiling of both β-artemether and lumefantrine as well as *in-silico* toxicity profile of lumefantrine and its related impurities have been reported in previous publications [20,23,28]. Here, *in-silico* toxicological profile of β-artemether was compared against its related identified impurities using Derek Nexus®. Derek Nexus® is a computer-based expert system for the qualitative prediction of possible toxic action of compounds on the basis of their chemical structure. The system is able to perceive chemical substructures within molecules and relate these to a rule-base, linking the substructures with likely types of toxicity [31]. The toxicity profile of β-artemether and all its identified related degradants and synthetic impurities is defined by several general toxicity alerts for mutagenicity, chromosomal abrasion, genotoxicity, skin irritation, hepatotoxicity and nephrotoxicity. However, no significant difference in *in-silico* toxicity profile between β-artemether and its related impurities is observed, which is also consistent with experimentally obtained Ames mutagenicity results [20].

## Conclusions

A stability-indicating HPLC method for simultaneous assay of β-artemether and lumefantrine fixed dose combination anti-malarial products was developed, using a fused-core reversed-phase amide stationary phase combined with an isocratic acetonitrile sodium phosphate mobile phase [Acetonitrile/1 mM phosphate buffer pH 3.0 (52:48, v/v)]. It is a rapid (four minutes total run time), precise and accurate method that can be utilized to quantify these anti-malarials in the presence of their related degradation products or impurities produced during inadequate transportation and storage. This method can be applied in the routine regulatory quality control of β-artemether and lumefantrine containing FDC drug products. The in-silico toxicological investigation using Derek Nexus® indicated overall a toxicity risk for β-artemether-related impurities comparable to that of the API β-artemether itself.

## Competing interests

The authors declare that they have no competing interests.

## Authors' contributions

SS executed the analytical laboratory experiments and, together with KV, wrote the manuscript. KV, MD and NB performed part of the analytical experiments and the *in-silico* toxicity evaluation. EW performed the statistical data analysis. LD, KP and CB critically reviewed this manuscript. BDS was the overall study director, responsible for experimental design, interpretation of data and review of the manuscript. All authors read and approved the final manuscript.

## Supplementary Material

Additional file 1: Figure S1 Chromatogram obtained on H_2_O_2_ stressed lumefantrine solution, with the PDA spectrum of the degradant peak eluting at RT 3.1min. **Figure S2**: Chromatogram obtained on acid stressed lumefantrine solution, with the PDA spectrum of the degradant peak eluting at RT 1.3min. **Figure S3**: Chromatogram obtained on dry heat stressed β-artemether solution at 210 nm. Click here for file
