# Supplementary material for: A rapid stability-indicating, fused-core HPLC method for simultaneous determination of β-artemether and lumefantrine in anti-malarial fixed dose combination products
Source: Malar J. 2013 Apr 30;12:145. doi: 10.1186/1475-2875-12-145 (PMC3651282; doi:10.1186/1475-2875-12-145)
Supplement: Additional file 1: Figure S1 — Chromatogram obtained on H2O2 stressed lumefantrine solution, with the PDA spectrum of the degradant peak eluting at RT 3.1min. Figure S2: Chromatogram obtained on acid stressed lumefantrine solution, with the PDA spectrum of the degradant peak eluting at RT 1.3min. Figure S3: Chromatogram obtained on dry heat stressed β-artemether solution at 210 nm. [file 1475-2875-12-145-S1.doc]

**Supplementary information**

1. **Figures**


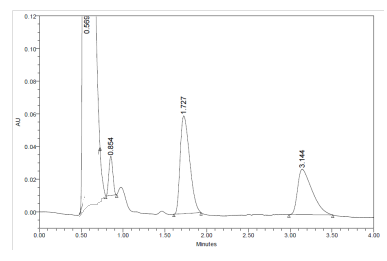


**Figure S1: Chromatogram obtained on H_2_O_2_ stressed lumefantrine solution, with the PDA spectrum of the degradant peak eluting at RT 3.1min.**

**Figure S2: Chromatogram obtained on acid stressed lumefantrine solution, with the PDA spectrum of the degradant peak eluting at RT 1.3min.**

**Figure S3: Chromatogram obtained on dry heat stressed β-artemether solution at 210 nm.**

1. **Formulas used for result calculations**

The asymmetry factor (*A_s_*) and retention factor (*k’*) were calculated based on Ph. Eur. as follows.

Where

- w_0.05_: the width of the peak at one-twentieth of the peak height, and
- d: distance between the perpendicular dropped from the peak maximum and the leading edge of the peak at one-twentieth of the peak height.


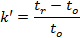


Where

- t_r_: retention time of the peak corresponding to the component., and
- t_o_: hold-up time corresponding to unretained component, usually the solvent.

β-artemether and lumefantrine concentrations were determined as per the formula given below and relative standard deviations (RSD) were calculated.


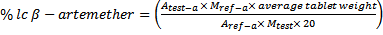


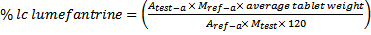


Where:

- A_test-a_: Area obtained for β-artemether on the test sample solution
- M_ref-a_: Mass of β-artemether reference standard
- A_ref-a_: Area obtained for β-artemether in the reference standard solution
- M_test_: Mass of the test sample tablet powder taken
- A_test-lum_: Area obtained for lumefantrine on the test sample solution
- M_ref-lum_: Mass of lumefantrine reference standard
- A_ref-lum_: Area obtained for lumefantrine in the reference standard solution

1. **Placket-Burman design and results**

**Table 1: Plackett-Burma**n design experiments and the varied parameters.

| ***#*** | ***Experiment Name*** | ***Parameters*** | | | |
| --- | --- | --- | --- | --- | --- |
|  |  | ***Flow rate, ml/min*** | ***% Acetonitrile in mobile phase*** | ***Mobile phase pH*** | ***Column temperature (˚C)*** |
| 1 | N1 | 1.2 | 50 | 2.0 | 35 |
| 2 | N2 | 1.2 | 54 | 2.8 | 25 |
| 3 | N3 | 1.2 | 54 | 3.2 | 25 |
| 4 | N4 | 0.8 | 54 | 3.2 | 35 |
| 5 | N5 | 1.2 | 50 | 3.2 | 35 |
| 6 | N6 | 0.8 | 54 | 2.8 | 35 |
| 7 | N7 | 0.8 | 50 | 3.2 | 25 |
| 8 | N8 | 0.8 | 50 | 2.8 | 25 |
| 9 | N9 | 1.0 | 52 | 3 | 30 |
| 10 | N10 | 1.0 | 52 | 3 | 30 |
| 11 | N11 | 1.0 | 52 | 3 | 30 |

**Table 2: Results** summary of robustness test experiments.

| ***Experiment name*** | ***As, Lumefantrine*** | ***k'*** | | ***%Content*** | |
| --- | --- | --- | --- | --- | --- |
|  |  | ***β-artemether*** | ***Lumefantrine*** | ***β-artemether*** | ***lumefantrine*** |
| N8 | 2 | 9.9 | 4.9 | 102.3 | 100.3 |
| N5 | 1.9 | 5.9 | 3.2 | 100.4 | 100.1 |
| N1 | 1.8 | 5.9 | 2.8 | 101.1 | 99.3 |
| N4 | 1.4 | 7.1 | 3.1 | 99.2 | 99.5 |
| N10 | 1.3 | 6.6 | 2.6 | 100.4 | 99.9 |
| N9 | 1.3 | 6.5 | 2.5 | 99.9 | 99.5 |
| N6 | 1.4 | 7.1 | 2.7 | 100.4 | 99.4 |
| N7 | 2.1 | 9.8 | 5.3 | 102.0 | 99.8 |
| N11 | 1.4 | 6.6 | 2.5 | 100.2 | 99.0 |
| N2 | 1.2 | 4.7 | 1.7 | 101.8 | 99.3 |
| N3 | 1.3 | 4.7 | 1.9 | 102.2 | 100.3 |
